# 

**Published:** 1919-05

**Authors:** 


### CONTENTS FOR THE MAY ISSUE

Health Resorts and Summer Vacations....371
Overworking....373
Sleeping Sickness....374
Auto-Intoxication....375
The New Sleeping Sickness....378
The Texas State Medical Meeting....384
Medical Letter-Bag....387
Medical Miscellany....395
New Advertisers and Subscribers....399
Book Reviews....400
Abstracts and Selections....401
Personal Mention....402
Helpful Hints....404
Old Jokes We Have Met....407

